# Every nurse an AI nurse: A framework for integrating artificial intelligence across nursing practice, education, research and policy

**DOI:** 10.1177/20552076251377939

**Published:** 2025-09-29

**Authors:** Mark Dornan

**Affiliations:** 1 School of Nursing and Midwifery, 1596 Queen's University Belfast, Belfast, UK

**Keywords:** Artificial intelligence, digital health, nursing practice, nursing leadership, nursing education

## Abstract

As artificial intelligence (AI) becomes increasingly embedded in healthcare, the nursing profession must embrace the imperative of ‘every nurse an AI nurse’. This commentary argues that nurses must not only adapt to AI technologies but actively shape their development, implementation, and governance to ensure alignment with nursing's core values of compassionate, patient-centred care. Drawing on a five-part APDDS framework: Aware, Prepare, Dare, Declare, Share; the article outlines a strategic approach for integrating AI across nursing practice, education, research, and policy. It emphasises the need for AI literacy, leadership, ethical engagement, and knowledge dissemination to empower nurses as innovators and advocates in the digital transformation of healthcare. By embracing this proactive stance, the nursing profession can ensure that AI enhances rather than diminishes the human elements of care.

## Introduction

In 2018, the Royal College of Nursing (RCN) in the United Kingdom (UK) declared ‘every nurse an e-nurse’,^1^ highlighting the need for nurses to embrace digital health technologies in an increasingly digital healthcare environment. Fast forward to 2025, and artificial intelligence (AI) has become a transformative force in healthcare, influencing not only patient care but also education, workforce dynamics, and research.

This evolution demands an even broader vision: ‘every nurse an AI nurse’. This is not merely a slogan, it is a critical imperative. AI is no longer a distant concept; it has become embedded in everyday, modern healthcare, offering vast potential to improve patient outcomes, enhance nursing workflows, and advance health equity,^2^ both for patients and nursing professionals. However, if nurses are not actively engaged, there is a risk that AI could exacerbate some of those inequities and diminish the core humanistic values at the centre of nursing.^3^

**Figure 1.**
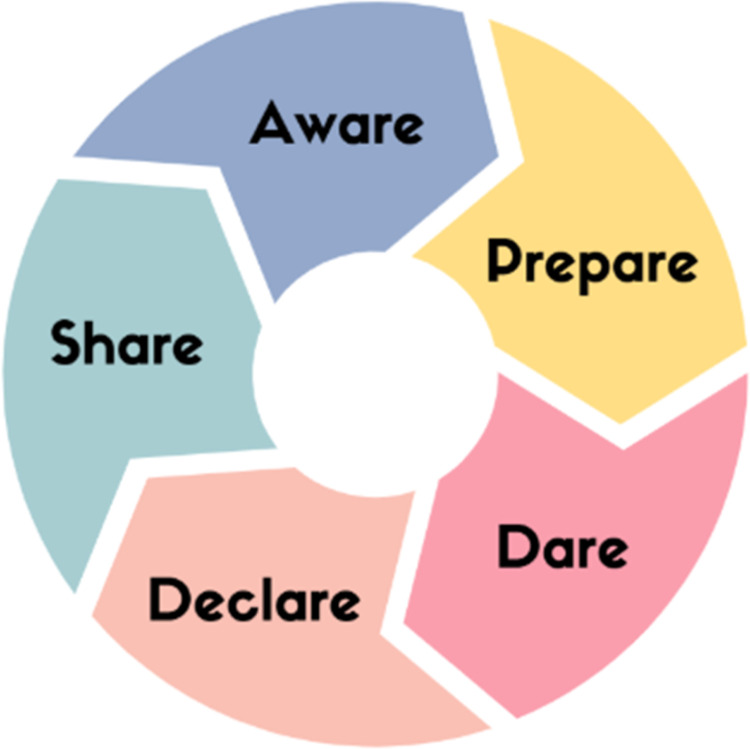
APDDS framework.

This commentary explores how ‘every nurse an AI nurse’ must be realised in practice, education, research, policy, and in collaborative working, ensuring AI aligns with nursing's commitment to compassionate, patient-centred care. To support this imperative, nurses must not only recognise the presence of AI but also actively shape its trajectory in health environments. Realising the vision of ‘every nurse an AI nurse’ requires more than passive adoption, it calls for a proactive and strategic approach that positions nurses as leaders and innovators in digital health. To guide this engagement, the author proposes a five-part APDDS framework that encapsulates the essential domains of action: nurses must be **aware**, **prepare**, **dare**, **declare**, and **share** (Figure 1). This article is presented as a commentary and call to action, offering a conceptual roadmap for the nursing profession. The APDDS framework introduced here is intended as a strategic lens through which nurses can engage with AI.

This framework provides a structured approach for understanding and operationalising nursing's role in an AI-enabled future. Figure 1 illustrates the APDDS framework as a circular model, emphasising the dynamic and iterative nature of nurse engagement with AI. Each component: Aware, Prepare, Dare, Declare, Share, builds upon and reinforces the others, reflecting the continuous cycle of learning, leadership, and advocacy required to integrate AI meaningfully into nursing practice. Each component highlights a key area of focus (Table 1) to ensure nurses are not only prepared for this transformation but are also leading it.

**Table 1.** Overview of APDDS framework.

Aware	Nurses must cultivate a *well-informed understanding* of AI's growing presence in healthcare. This includes recognising the potential benefits, as well as the risks. Awareness is the first step toward responsible engagement.
Prepare	To navigate AI confidently and competently, nurses need access to robust *education* and *training*. This means embedding AI literacy into nursing curricula and offering ongoing professional development.
Dare	Nurses must be bold in shaping the future of healthcare with AI. This involves stepping into *leadership* roles, challenging assumptions, and advocating for ethical, equitable AI practices.
Declare	Nurses should actively participate in the strategic and *policy-level* decisions that guide AI implementation. Engagement in governance, from institutional planning to national policy development, ensures that nursing perspectives shape the broader AI landscape.
Share	Nurses must contribute to the growing body of evidence on AI in healthcare. By sharing experiences and outcomes from practice, and *dissemination* through research, publications, and interdisciplinary collaboration.

Each section is expanded upon below.

## Framework

### Aware

Nurses must first develop a clear understanding of the growing influence of AI in healthcare. No longer a futuristic concept, AI is already embedded in everyday clinical practice, supporting tasks such as documentation, triage, remote monitoring, and diagnostics. A 2024 survey reports that 66% of American physicians sampled, use some form of AI in their practice.^4^ Already in nursing, for example, predictive algorithms that flag patient deterioration and mental health chatbots are reshaping how nurses interact with both patients and healthcare systems.^5,6^

This awareness also extends to understanding how nursing data powers AI systems. Nurses generate vast amounts of clinical data, from electronic health records (EHRs) to observational notes, that often serve as the foundation for AI tools. Yet, despite being key data contributors, nurses are frequently excluded from the development, validation, implementation and evaluation of these technologies.^7^ This lack of involvement can result in tools that miss clinical nuance, reinforce bias, or fail to reflect the realities of patient care.

New evidence is beginning to indicate the urgency of building AI awareness among nurses. Starr et al.^8^ note that 70% of US nurses in 2021 had little to no knowledge of AI, reflecting a significant gap in foundational understanding. However, in a recently published study, Atalla et al.^9^ found in a cross-sectional study, that nurses demonstrated high levels of AI-related acceptance attitudes, highlighting a rapid change in the profession's capacity to adapt to new technology. Importantly, the study emphasised that integrating AI-focused content into nursing education and promoting continuous professional development are critical strategies to strengthen readiness for AI-driven healthcare practice.

To understand the current rise in AI adoption, it's important to recognise earlier work. Topaz and Pruinelli,^10^ outlined how ‘big data’, and emerging technologies could shape nursing but also drew attention to AI's roots in the profession back to the 1980s. Their work highlighted both the promise of AI in clinical decision-making and challenges such as limited training, EHR usability, and data governance. These remain relevant and applicable challenges. Early applications, like Natural Language Processing (NLP) for clinical notes and predictive models for post-acute care referrals,^11^ demonstrated the value of data-driven nursing. Though predating generative AI, these contributions remain relevant, reinforcing the need for interdisciplinary collaboration and nursing-specific AI competencies.

Moreover, global digital inequality must be acknowledged. The World Health Organization^12^ has raised concerns about ‘data colonialism’, where AI technologies developed in the Global North are deployed in other regions, rural and low-income countries, without sufficient adaptation. In this context, awareness also means recognising the socio-political dimensions of AI and its potential to exacerbate health disparities if not implemented with cultural and contextual sensitivity.

### Prepare

Preparing nurses for the age of AI requires both formal education and ongoing professional development. Despite the growing presence of AI in healthcare, many nursing programmes still lack dedicated content on this topic. While 59.9% nursing students were aware of AI in healthcare in one study, they reported a lack of available educational resources (35.8%) and mentorship (41.4%).^13^ Graduates often enter the workforce without the foundational knowledge needed to engage confidently with AI technologies; however, AI has a provide a powerful opportunity to transform nursing education.^14^ Integrating AI into undergraduate curricula, covering how algorithms function, what data they rely on, and how to interpret their outputs is essential for the future nursing education and practice. For example, generative AI tools like ChatGPT are being used by students to brainstorm research questions and refine literature reviews.^15^ While these tools offer new opportunities for learning, they also raise concerns about academic integrity and the accuracy of generated content.^16^ To address these challenges, Gosak et al.^15^ advocate for careful consideration on the over-reliance and ethical AI use across all levels of nursing education.

Beyond initial training, continuing professional development plays a key role in building confidence and competence, across all professional levels.^7^ Nurse educators themselves need support to stay current with AI's evolving capabilities and its’ limitations, however, will require support to achieve this.^17^ AI education must be seen as both horizontal and vertical. Horizontally, it should not be seen as stand-alone learning, rather, embedded across curricula content, such as: acute care management, chronic health management, pharmacology, leadership and management, research, specialist practice – from oncology to cardiology and intensive care to primary care. Vertically, AI content needs to start at introductory-level understanding and move through advancing levels towards proficiency, competency and confidence.

Digital leadership and informatics competencies should be embedded in postgraduate and specialist training. Nurses in leadership or informatics roles must be equipped to critically evaluate AI tools and guide their responsible implementation in clinical settings. As Charow et al.^18^ emphasise, future education efforts should take a multidisciplinary and competency-based approach to curriculum redesign, focusing not only on technical skills but also on regulatory strategies and the preservation of patient–clinician interaction. By investing in education and training, we empower nurses to use AI tools safely, ethically, and effectively, ensuring they can lead, not merely adapt to, the digital transformation of healthcare.

### Dare

To ‘dare’ in the age of AI is to embrace leadership and innovation in shaping how emerging technologies are integrated into healthcare. Nurses have historically been underrepresented in digital health leadership, often positioned as end-users rather than co-creators. Addressing this imbalance requires both structural reform and a cultural shift, nurses must be empowered to act as designers, evaluators, and decision-makers in AI development. Leadership also involves advocating for core nursing values, such as holistic, person-centred care within interdisciplinary AI teams. Collaboration between nurses, data scientists, engineers, and patients is essential to ensure that AI tools are clinically relevant, ethically sound, and acceptable to those who use them. Tarsuslu et al.^19^ demonstrate that strong digital leadership can transform AI-related anxiety into proactive engagement, fostering more positive attitudes toward AI adoption in nursing. Equally, a qualitative study indicated that nurse leaders could see the benefits that AI would bring but recognised the challenges of organisational readiness, support and culture as potential barriers.^20^ These values were shared by Rony et al.^21^ where nurses spoke of their collective preparedness and optimism of AI in nursing care.

Innovation is already underway. For example, AI-enhanced wound assessment tools are being developed to support more accurate and efficient care delivery, with promising applications in clinical decision-making and documentation.^22^ These technologies, when co-designed with nurses, not only streamline routine tasks but also enhance the quality and safety of patient care. To sustain this momentum, healthcare organisations must create environments that support and reward innovation. This includes providing protected time for digital experimentation, offering seed funding for nurse-led AI projects (such as AI Nurses Network^23^), and formally recognising contributions to technological advancement. By daring to lead, innovate, and advocate, nurses can ensure that AI evolves in ways that enhance, rather than replace the human elements of care.

### Declare

Nurses must play a visible and active role in the governance and policymaking processes that shape how AI is integrated into healthcare. This includes contributing to strategic planning, procurement decisions, and the development of local implementation frameworks. Without nursing input, policies risk being disconnected from frontline realities, potentially leading to poor adoption and even patient safety concerns. There is growing momentum for professional bodies, such as the RCN and others, to lead the development of national guidelines on AI in healthcare. These organisations must convene diverse nursing voices to co-create position statements, protocols, and educational standards that reflect the profession's values and practical needs. Participation in these processes is not optional; it is a professional responsibility.

Nurses, as the one of the largest health professionals, should also be represented in ethics reviews and, algorithm evaluation and implementation committees. Their clinical insight is essential to ensure that AI tools are safe, usable, and contextually appropriate. Effective governance also requires clarity around accountability: who is responsible when AI influences clinical decisions, and how systems will log, audit, and respond to AI-related adverse events. Initiatives such as BE FAIR (Bias Elimination for Fair and Responsible AI) highlight the importance of inclusive oversight. As Cary et al.^24^ argue, fairness must be embedded as an operational principle, not just a theoretical goal, and nurses are critical to making that principle a reality in practice.

While the APDDS framework was developed with broad applicability in mind, its successful implementation will vary across different global contexts. In low- and middle-income countries or under-resourced health systems, considerations such as digital infrastructure, workforce capacity, and access to education must shape how each component is prioritised.^25^ Awareness and education may require tailored, locally led approaches using low-cost, and integration into existing infrastructure that complement existing processes and technology.^26^ Similarly, declarations of governance may need to align with regional policies or international support structures. Recognising these contextual variations is essential for ensuring equitable AI integration and preventing the amplification of global health disparities. With the development and validation of new and specific research evaluation scales pertaining to AI and nursing, more nuanced, specific data will become available to tailor education and skills development according to environment.^27^

### Share

Sharing knowledge, experiences, and research findings is essential to advancing nursing's role in the development and governance of AI. While many nurses are now engaging with AI tools in clinical settings, relatively few are publishing evaluations or sharing insights through formal academic or professional channels. This limits the profession's ability to learn collectively, refine best practices, and influence the direction of AI innovation.

However, In January 2025, the Journal of Nursing Scholarship published a special issue titled ‘Transformative Role of Artificial Intelligence in Nursing’, which showcased the growing scholarly engagement of nurses with AI. The issue featured empirical studies led by nurses,^28,29^ critical reviews of AI applications in clinical practice,^30,31^ and discussions of ethical challenges and governance.^19,22^ Collectively, these contributions reflect a shift from the vision of ‘every nurse an e-nurse’ to the emerging reality of ‘every nurse an AI nurse’.

Research on AI in nursing should embrace both qualitative and quantitative approaches. Studies can explore how AI affects clinical decision-making, task delegation, patient interaction, and nurse wellbeing, including burnout. There are also growing opportunities for interdisciplinary collaboration. For example, Chae et al.^32^ developed a clinical decision support framework that integrates predictive modelling into routine nursing practice in home health care for patients with heart failure, offering good ground for nurse-led research and innovation. Importantly, sharing should extend beyond academia. Informal peer learning and webinars can help disseminate knowledge within healthcare organisations. Creating communities of practice, whether locally and national to collectively address common issues, support innovation, and strengthen professional networks. By actively sharing insights and evidence, nurses can shape the future of AI in healthcare, ensuring it remains grounded in clinical realities and nursing values.

## Conclusion

The call for ‘every nurse an AI nurse’ marks a shift in how the nursing profession must engage with AI. The APDDS Framework offers a structured approach to guide this transformation, ensuring that nurses are equipped to lead, not just adapt to, the integration of AI in healthcare. Nurses must become active participants in AI's development and governance, as designers, evaluators, educators, and advocates. Their leadership is essential to ensure AI supports clinical judgment, promotes equity, and can enhance modern-day patient-centred care. With the right vision and support, nurses can shape a future where AI strengthens, rather than replaces, the human elements of healthcare.
